# Synergistic Activation of Bovine CD4+ T Cells by Neutrophils and IL-12

**DOI:** 10.3390/pathogens10060694

**Published:** 2021-06-03

**Authors:** Zhengguo Xiao, Anmol Kandel, Lei Li

**Affiliations:** Department of Animal and Avian Sciences, University of Maryland, College Park, MD 20742, USA; akandel1@umd.edu (A.K.); lixxx242@umd.edu (L.L.)

**Keywords:** cattle, CD4+ T cells, neutrophils, CD28, IL-12, three signals, activation

## Abstract

CD4+ T cell activation requires inflammatory cytokines to provide a third signal (3SI), such as interleukin-12 (IL-12). We recently reported that bovine neutrophils can enhance the activation of bovine CD4+ T cells. To explore the interactions between neutrophils and third signal cytokines in bovine CD4+ T cell activation, naïve CD4+ T cells were isolated from cattle lymph nodes and stimulated for 3.5 days with anti-bovine CD3 (first signal; 1SI), anti-bovine CD28 (second signal; 2SI), and recombinant human IL-12 (3SI) in the presence or absence of neutrophils harvested from the same animals. Indeed, the strongest activation was achieved in the presence of all three signals, as demonstrated by CD25 upregulation, IFNγ production in CD4+ T cells, and secretion of IFNγ and IL-2 in cell supernatants. More importantly, 1SI plus neutrophils led to enhanced CD25 expression that was further increased by IL-12, suggesting synergistic action by IL-12 and neutrophils. Consistently, neutrophils significantly increased IFNγ production in 1SI plus IL-12-stimulated CD4+ T cells. Our data suggest the synergy of neutrophils and IL-12 as a novel regulator on bovine CD4+ T cell activation in addition to three signals. This knowledge could assist the development of immune interventions for the control of infectious diseases in cattle.

## 1. Introduction

CD4+ T cells contribute to adaptive immunity against pathogens in animals by helping B cells to produce pathogen-specific antibodies, including neutralizing antibodies [1,2], and enhance the killing capacity of CD8+ T cells against pathogen-infected or malignant cells, and are instrumental in the induction of CD8+ T cell memory after vaccination [3,4,5]. Naïve CD4+ T cells require specific signaling for full activation via antigen-specific receptors and costimulation from dendritic cells (DCs) in the draining lymph nodes (DLNs) [6]. Cytokines further direct CD4+ T cell differentiation into specific subsets, such as IL-12 for Th1, and IL-4 for Th2 [1,7].

In humans and mice, full activation of naive CD4+ T cells depends on three signals: antigen (1SI), costimulation (2SI), and third signal cytokines (3SI) [8]. Professional antigen-presenting cells (APCs) provide these signals via expression of MHC II, which presents pathogen-derived peptides to naive CD4+ T cells, and the expression of costimulatory molecules and production of cytokines to determine the type of response required [9,10,11]. However, it is not clear if bovine naive CD4+ T cells are activated and directed in the same manner.

Costimulation from professional APCs can deliver stimulatory or inhibitory effects to T cells and include members of the immunoglobulin superfamily (IgSF) and tumor necrosis factor receptor superfamily (TNFRSF) [8,12]. Each superfamily is subdivided according to its stimulatory or inhibitory activity [8]. The most studied stimulatory 2SI molecules are CD28 and B7 of the IgSF, and CD40-CD40L of the TNFRSF [8]. Typical inhibitory 2SI molecules include CTLA-4, PD-1L, LAIR1, TIM3, and TIGIT, which participate in the establishment of chronic infection and cancers [13,14,15,16]. 

Neutrophils, also known as polymorphonuclear leukocytes, are produced in abundance in mammals and are the predominant leukocyte circulating in the blood. Neutrophils make up 50–70% of circulating white blood cells and provide the first line of defense against pathogens and injury [17,18,19]. Neutrophils tend to be short-lived effector cells with circulating lifespans of about 8 hours in humans [20,21], although some studies have demonstrated that they can survive for at least a day [22,23,24]. Neutrophils can quickly (in hours) migrate into draining lymph nodes after pathogen challenges or vaccination and can efficiently prime CD8+ T cells in mice [25,26,27,28]. In addition, neutrophils are detected in secondary lymphoid tissues in healthy humans and mice, including lymph nodes [29]. Similarly, we have found neutrophils in lymph nodes and the spleen from clinically healthy cattle, as well as from parasite-challenged calves [30]. Recently, neutrophils were reported to enhance CD3-driven activation of naïve bovine CD4+ T cells, without the requirement of additional costimulation [30]. These data suggest that bovine neutrophils may work together with cytokines in the activation of bovine CD4+ helper T cells. 

In this report, we tested the function of the third signal cytokine IL-12 in a three-signal model on bovine CD4+ T cell activation, using anti-CD3 to provide TCR signaling, anti-CD28 for the second signal and IL-12 as the third signal. More specifically, we examined the activation of bovine CD4+ T cells by bovine neutrophils together with IL-12. We identified a unique synergy of neutrophils and IL-12 on bovine CD4+T cell activation, suggesting new strategies of immune intervention targeting activation of CD4+ T cells in cattle.

## 2. Results

### 2.1. Bovine CD4+ T Cell Activation Requires Third Signal Cytokine 

**Figure 1:**
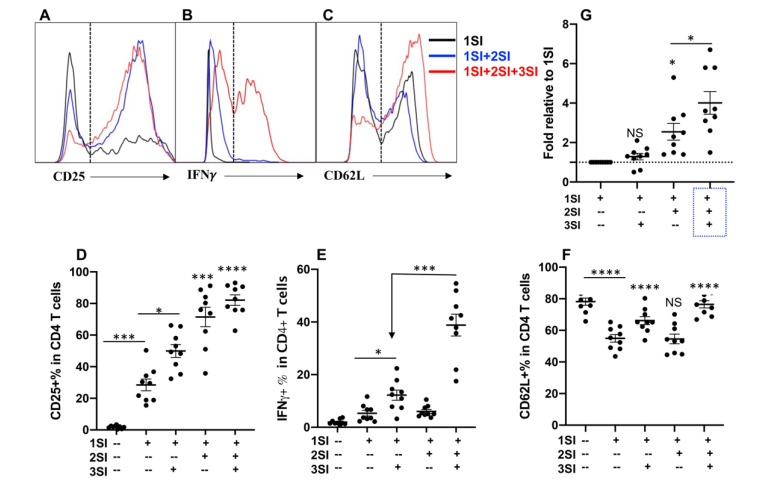
Bovine CD4+ T cell activation requires third signal cytokine. Sorted naïve CD4+ T cells from three cattle were stimulated for 3.5 days with different combinations of anti-bovine CD3 antibody (αCD3 for 1SI), anti-bovine CD28 antibody (αCD28 for 2SI) and recombinant human IL-12 (IL-12 for 3SI). Cells and supernatants were harvested for analysis after 3.5 days. (**A**–**C**) Gating strategy for CD25 (A), IFNγ (B), and CD62L (C). (**D**–**F**) Comparison of expression of CD25 (D), IFNγ (E), and CD62L (F) in CD4+ T cells. Each dot represents one animal. (**G**) Fold changes of cell numbers relative to 1SI. Data were pooled together from three experiments, and expressed as mean of the nine cattle samples with standard error of the mean (SEM). All data were analyzed by one-way ANOVA with Tukey’s Multiple Comparisons Test. Asterisks indicate statistical significance. * *p* < 0.05; *** *p* < 0.001; **** *p* < 0.0001. NS: not significant.

Anti-bovine CD3 was used to provide first signal (1SI), anti-bovine CD28 as second signal (2SI). Recombinant human IL-12 has been shown to effectively stimulate bovine natural killer (NK) cells [31,32], and thus was used to provide third signal (3SI). Naïve CD4+ T cells were isolated and sorted from inguinal lymph nodes (Supplementary Figure S1), and stimulated with different combinations of signals for 3.5 days, as reported previously [30]. Consistent with data from humans and mice [8], the presence of all of the three signals (1SI+2SI+3SI) led to the strongest activation, indicated by the highest level of expression of CD25 and IFNγ in CD4+ T cells (Figure 1A,B,D,E), and the highest cell expansion (Figure 1G). Although 2SI enhanced the expression of CD25 in 1SI-stimulated bovine CD4+ T cells (Figure 1D), 2SI did not significantly affect IFNγ induction (Figure 1E). On the other hand, 3SI (IL-12) was potent on IFNγ induction together with 1SI, and the presence of 2SI significantly potentiated this function of 3SI (Figure 1E), suggesting that bovine CD4+ T cells respond to three signals in a way similar to CD4+ T cells in humans and mice [8]. CD62L is usually expressed at high level in naïve CD4+ and CD8+ T cells, which plays a pivotal role in controlling naïve T cells to traffic to and from secondary lymphoid tissues [10]. Once T cells are exposed to antigen stimulation in lymph nodes, the expression of CD62L decreases, which enable the activated T cells to migrate to the site of infection to perform their functions. Consistent with their human and murine counterparts [5,33]. CD62L in bovine CD+ T cells was significantly downregulated by anti-CD3 stimulation (1SI) (Figure 1C,F). However, IL-12 enhanced the expression of CD62L irrespective of 2SI (Figure 1F), suggesting CD62L expression in bovine CD4+ may be sensitive to IL-12 regulation, similar to the effects of IL12 on CD4+ and CD8+ T cells in mice [34,35].

### 2.2. Bovine Naïve CD4+ T Cell Activation Leads to Cytokine Secretion 

**Figure 2:**
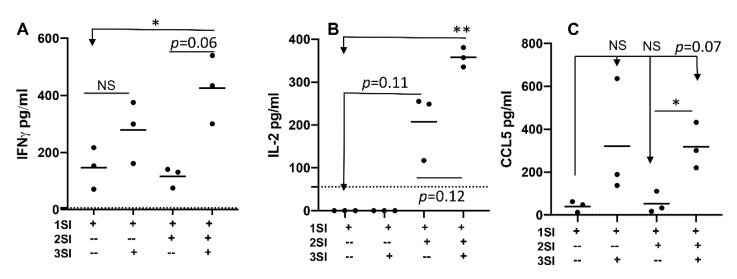
Activation of bovine naïve CD4+ T cells leads to secretion of cytokines. Supernatants from stimulated CD4+ T cells were harvested 3.5 days after stimulation and quantified for cytokine IFNγ (**A**), IL-2 (**B**), and chemokine CCL5 (**C**), using a cytokine array [36,37]. Data were expressed as mean of three cattle samples and were analyzed by one-way ANOVA with Tukey’s Multiple Comparisons Test. Asterisks indicate statistical significance. * *p* < 0.05; ** *p* < 0.01. NS: not significant. Dotted lines indicate limit of detection (LOD) for each cytokine.

Cytokines need to be secreted by cytokine producing CD4+ T cells, which bind to their corresponding receptors on targeted cells. We examined whether cytokine secretion patterns were altered with activation status of bovine CD4+ T cell, by quantifying supernatant cytokine profiles using a bovine cytokine array (RayBiotech Inc., Peachtree Corners, GA). Indeed, IFNγ and IL-2 were detectable at the highest level when all three signals were present, whereas omission of either 2SI or 3SI reduced their secretion (Figure 2A,B). Like Figure 1D, 3SI seemed to increase IFNγ secretion in 1SI-stimulated CD4+ T cells, but not significant (Figure 2A), whereas secretion of IL-2 was more associated with 2SI than with 3SI (Figure 2B). Chemokine CCL5 was detected in supernatants exposed to 3SI+1SI, and was at a significantly higher concentration when all three signals were present compared with 1SI+2SI (Figure 2C). These data indicated that activation of bovine CD4+ T cells with all three signals leads to not only strong production, but also strong secretion of cytokines, including IFNγ and IL2, and chemokines like CCL5. 

### 2.3. Neutrophils and IL-12 Synergistically Activate Bovine CD4+ T Cells 

**Figure 3:**
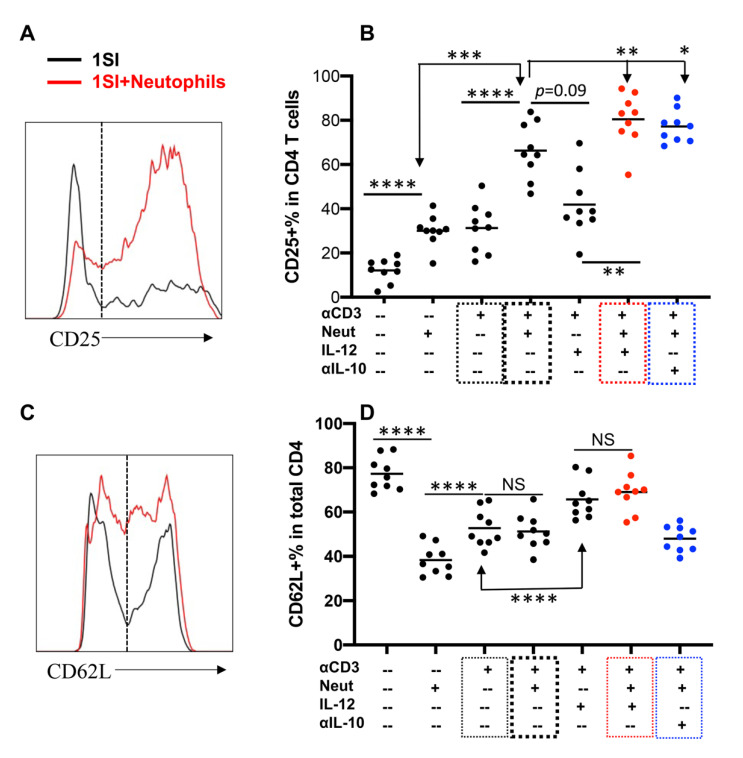
Neutrophils and IL-12 are synergistic on activation of bovine CD4+ T cells. Sorted naïve CD4+ T cells were stimulated for 3.5 days with different combinations of αCD3, IL-12, neutrophils (Neut), and anti-bovine IL-10 neutralizing antibody (αIL-10). Cells and supernatants were harvested for analysis after 3.5 days. (**A**) CD25 gating strategy. (**B**) Comparison of % of CD25+ in CD4+ T cells. (**C**) CD62L gating strategy. (**D**) Comparison of % of CD62L+ in CD4+ T cells. Dotted boxes indicated treatments for comparison. Thin black: αCD3 only; thick box: Neutrophils+αCD3; red box: Neutrophils+αCD3+IL-12; blue box: Neutrophils+αCD3+αIL-10. Data were pooled together from three experiments, and expressed as mean of the nine cattle samples with standard error of the mean (SEM). All data were analyzed by one-way ANOVA with Tukey’s Multiple Comparisons Test. Asterisks indicate statistical significance. * *p* < 0.05; ** *p* < 0.01; *** *p* < 0.001; **** *p* < 0.0001. NS: not significant.

We recently reported that bovine neutrophils could activate bovine CD4+ T cells together with 1SI and precluded the requirement for additional costimulation [30]. The potential synergistic effects of neutrophils and IL-12 were accessed by stimulating bovine CD4+ T cells with 1SI in the presence or absence of IL-12 and/or neutrophils in different combinations. The presence of neutrophils enhanced CD25 expression in 1SI-stimulated gated CD4+ T cells (Supplementary Figure S3, Figure 3A,B, thick black box), consistent with our previous report [30]. The presence of both neutrophils IL-12 led to significant upregulation of CD25 in 1SI-stimulated CD4+ T cells compared to that of either alone (Figure 3B, red box), suggesting synergistic activity by IL-12 and neutrophils. Consistently, IL-12 enhanced CD62L expression only in the presence of neutrophils (Figure 3C,D). In addition, the presence of neutrophils, with or without IL-10, did not affect the percentage of CD62L expressing cells (Figure 3D, blue box), suggesting that the synergy between neutrophils and IL-12 is restricted to CD4+ T cell activation and may not necessarily affect their migration.

### 2.4. Neutrophils and IL-12 Synergistically Stimulate the Secretion of Cytokines by Activated Bovine CD4+ T Cells 

**Figure 4:**
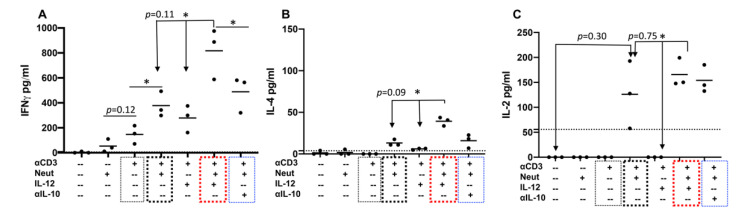
Neutrophils and IL-12 synergistically stimulate the secretion of cytokines by activated bovine CD4+ T cells. Supernatants from stimulated CD4+ T cells were harvested 3.5 days after stimulation and quantified for Th1 cytokine IFNγ (**A**), Th2 cytokine IL-4 (**B**), plus IL-2 (**C**), using a cytokine array [36,37]. Thin black: αCD3 only; thick box: Neutrophils+αCD3; red box: Neutrophils+αCD3+IL-12; blue box: Neutrophils+αCD3+αIL-10. Data were expressed as mean of three cattle samples and were analyzed by one-way ANOVA with Tukey’s Multiple Comparisons Test. Asterisks indicate statistical significance. * *p* < 0.05. NS: not significant. Dotted lines indicate limit of detection (LOD) for each cytokine.

Supernatants were harvested from activated cells and quantified for cytokine profiles using a bovine cytokine array. Like CD25 regulation in Figure 3B, the simultaneous presence of neutrophils and IL-12 significantly increased the secretion of IFNγ to a greater degree than IL-12 together with 1SI, but not significant compared with neutrophils+1SI (*p* = 0.11) (Figure 4A, red box). The depletion of IL-10 from neutrophil coculture enhanced IFNγ secretion, but not significantly (Figure 4A, blue box). IL-4 was detectable in the supernatants of neutrophils+1SI and was increased by IL-12 but not significantly (*p* = 0.09) (Figure 4B, thick black box and blue boxes). Similarly, IL-4 was barely detectable in that of 1SI+IL-12, and was significantly enhanced when neutrophils were added (Figure 4B). Consistent with these data, low levels of IL-4-producing cells were also detected via intracellular staining (Supplementary Figure S4). Neutrophils induced detectable IL-2 in the presence of 1SI that was not significantly affected by additional IL-12 or IL-10 depletion (Figure 4C, red and blue boxes). These data confirmed the synergistic effects of neutrophils and IL-12 on cytokine secretion, consistent with their synergistic effects on CD25 expression (Figure 3B). 

### 2.5. CD28 Can Deliver Inhibitory Signaling to Bovine CD4+ T Cells 

**Figure 5:**
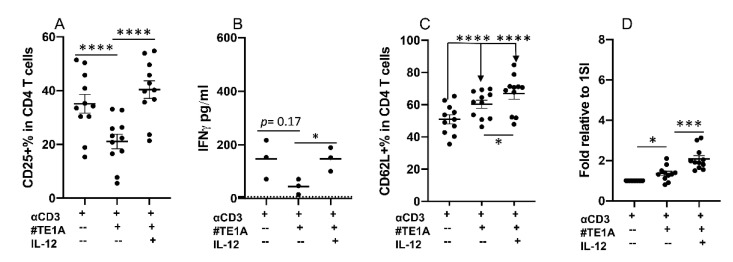
CD28 can deliver inhibitory signaling to bovine CD4+ T cells. Sorted naïve CD4+ T cells from three cattle were stimulated for 3.5 days with different combinations of αCD3, αCD28 mAb Clone#TE1A, and IL-12. Cells and supernatants were harvested for analysis after 3.5 days. **1SI**: αCD3. Comparison of % of CD25+ (**A**), quantification of IFNγ (**B**) and CD62L (**C**) in the supernatant and fold changes relative to 1SI (**D**) in CD4+ T cells. Dotted line in B indicated limit of detection. Data in A, C and D were pooled together from four experiments, and expressed as mean of the 11 cattle samples with SEM. Data in B were expressed as mean of three cattle samples. All data were analyzed by one-way ANOVA with Tukey’s Multiple Comparisons Test. Asterisks indicate statistical significance. * *p* < 0.05; *** *p* < 0.001; **** *p* < 0.0001. NS: not significant.

We examined costimulatory function of CD28 by experimenting a series of clones of anti-bovine CD28 monoclonal antibodies. To our surprise, one specific clone, Clone#TE1A, reduced CD25 expression in 1SI-stimulated bovine CD4+ T cells, which was rescued by IL-12 (Figure 5A). Similarly, IFNγ production was decreased (not significantly) by Clone#TE1A, and IL-12 restored the reduction (Figure 5B). Both CD62L expression and cell expansion were increased slightly but significantly by Clone#TE1A, which was further raised by IL-12 (Figure 5C,D). These data indicate that CD28 can deliver inhibitory signals to bovine CD4+ T cells.

### 2.6. Synergy between Neutrophils and IL-12 Can Be Regulated by Inhibitory Signaling from CD28 

**Figure 6:**
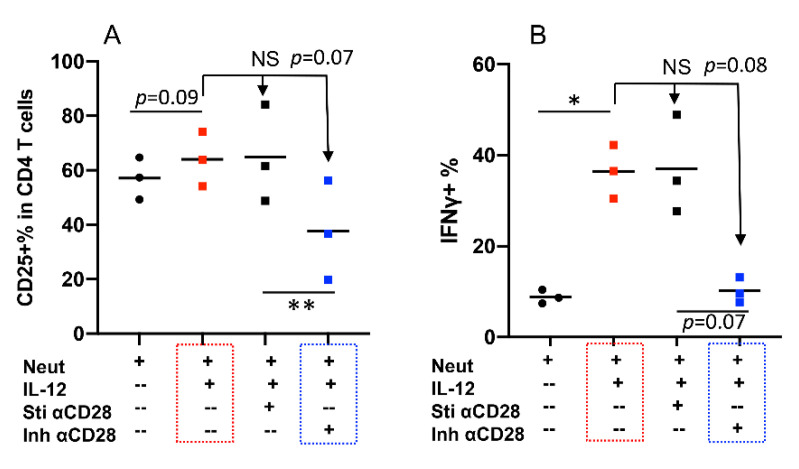
Inhibitory signaling delivered from CD28 dampens synergy of neutrophil and IL-12. Sorted naïve CD4+ T cells from three cattle were stimulated with αCD3 for 3.5 days under synergistic condition (neutrophils plus IL-12) in the presence or absence of stimulatory αCD28 mAb (Sti αCD28) and inhibitory αCD28 mAb (Inh αCD28). Comparison of % of CD25+ (**A**), IFNγ+ (**B**) in CD4+ T cells. Red box: Neutrophils+αCD3+IL-12; blue box: Neutrophils+αCD3+ αCD28 mAb (Inh αCD28). Data were expressed as mean of three cattle samples and were analyzed by one-way ANOVA with Tukey’s Multiple Comparisons Test. Asterisks indicate statistical significance. * *p* < 0.05; ** *p* < 0.01. NS: not significant.

Naïve bovine CD4+ T cells were stimulated by 1SI plus neutrophils with or without IL-12 in the presence or absence of stimulatory anti-CD28 (Sti αCD28) and inhibitory anti-CD28 clone#TE1A (Inh αCD28). Indeed, Clone#TE1A dampened neutrophil and IL12 synergy (*p* = 0.07), as shown by reduced CD25 expression and IFNγ production (*p* = 0.08), whereas stimulatory anti-CD28 (Sti αCD28) had essentially no effect (Figure 6A,B, red and blue boxes). These data suggest that the synergy of neutrophil and IL-12 can be regulated, such as through inhibition by specific clone of anti-bovine CD28 TE1A.

## 3. Discussion

**Figure 7:**
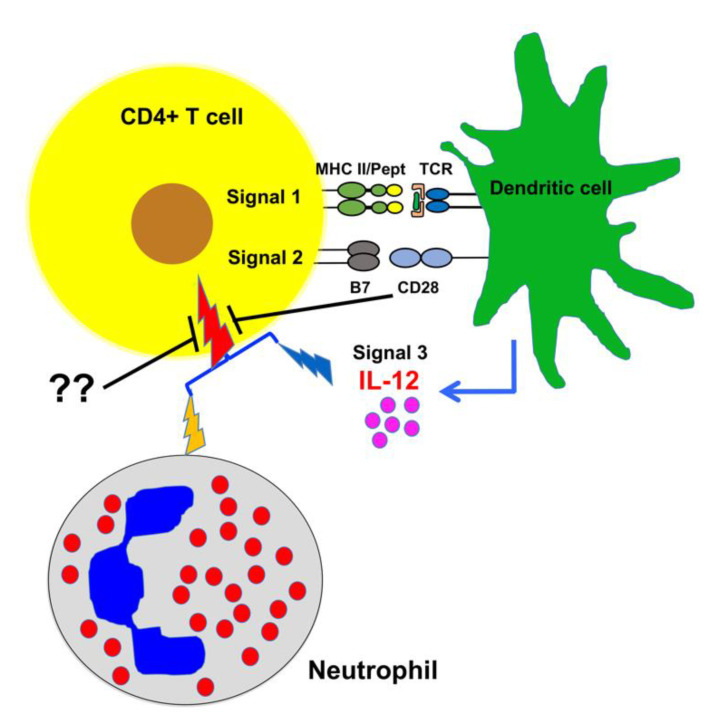
Synergy of neutrophils and IL-12 on the activation of bovine CD4+ T cells. Professional antigen-presenting cells like dendritic cells can provide all three signals to activate bovine CD4+ T cells. However, the presence of neutrophils can potentiate the function of IL-12, thus generates a synergy (red thunderbolt symbol) between neutrophil (yellow thunderbolt symbol) and IL-12 (blue thunderbolt). This synergy could be regulated by inhibitory signals, such as the one triggered by anti-bovine CD28 clone#TE1A.

As a linchpin for adaptive immunity, CD4+ T cells need to be activated to obtain effector functions, which requires three different signals. Similar to observed in humans and mice, we found that three signals together (antigen, costimulation and cytokine IL-12) give the strongest stimulation to bovine CD4+ T cells, compared to one or two signals. More importantly, bovine neutrophils enhance the activation of bovine CD4+ T cells driven by anti-bovine CD3, which is further increased by IL-12, demonstrating a synergy between IL-12 and neutrophils. This synergy can be regulated by inhibitory signaling on bovine CD4+ T cells, suggesting unique interplays between immune cells in cattle (Figure 7).

In general, dendritic cells (DCs) can provide all three signals to T cells in humans and mice [38,39]. CD28 costimulatory pathway has been thoroughly studied [40,41], and its costimulatory function to TCR signaling is similarly reflected in cattle as described in this report. However, the inhibitory effects from anti-CD28 Clone#TE1A is rather surprising. At this moment, we do not know any equivalent observation in mAb clones against CD28 in human and mice. CD28 and CTLA-4 are highly homologous, but they deliver opposite effects to T cells during activation [41]. We speculate that this Clone#TE1A may target an antigen epitope shared between CTLA-4 and CD28 in cattle T cells. CD28 and CTLA-4 compete for the same ligands CD80 and CD86 [42], and CTLA-4 binds these ligands with a higher affinity than CD28 does, which allows CTLA-4 to suppress effector T cell responses [43]. CTLA-4 binding to CD80 or CD86 is always stronger than CD28 binding when only a single ligand (either CD80 or CD86) is present. However, when both CD80 and CD86 are present, CD86 has a relative preference for CD28, while CD80 prefers binding to CTLA-4 [44]. Thus, it is possible the clone#TE1A may have functions similar to CD80, by binding to the CD80 binding site shared by CD28 and CTLA-4, which preferentially binds to CTLA-4 and thus triggers suppressive effects on bovine CD4+ T cells. CTLA-4 is expressed in activated T cells and regulator T cells in humans and mice [45,46]. Interestingly, this clone has been reported to be used together with anti-bovine CD3 to provide two signals [47], which is a common immunological assay. Clone#TE1A shows stimulatory function in antigen-specific IFNγ-based recall response in the PBMC from Mycobacterium avium subspecies paratuberculosis vaccinated cattle [48]. We have noticed that the requirements for reactivation may vary between naive vs. memory T cells [49]. Additionally, this stimulatory effect could also be contributed by indirect effects from other CD28-expressing cells in the PBMC [40,41]. At this moment, we cannot exclude the possibility that bovine CD28 molecule may not have the identical scope of function like its counterparts in humans and mice. It has been clearly shown that CD28 signaling pathway in regulatory T cell differentiation and activation is different from that in CD4+ and CD8+ T cells, which contribute more to anti-inflammatory status and homeostasis [40,41]. Furthermore, the effects of CD28 on T cells are complicated, and broad on spectrum at multiple levels including transcriptional and translational [40,41]. It will be very interesting to identify the binding site of TE1A clone compared to that of stimulatory clones, such as clone# CC219 [50], which is under investigation in our lab. 

Bovine CD4+ T cells are activated and differentiated to effector T cells, such as Th1/Th2 responses to pathogens similar to those in humans and mice [51,52]. Accordingly, the Th1 response has been associated with control of intracellular pathogens in cattle, such as bovine viral diarrhea virus [53,54,55,56], foot and mouth disease [57], Mycobacterium bovis [58,59], and Mycobacterium avium subspecies paratuberculosis (MAP) [60,61,62,63,64]. Elegant work on the establishment of bovine CD4+ T cell clones specific to epitopes derived from Babesia bigemina definitively shows Th1 protection against intracellular pathogens in cattle [65,66,67]. As in humans and mice, Th2 is induced by extracellular pathogen infections in cattle [68,69,70,71,72]. In animals other than humans and mice, however, the CD4+ T cell differentiation process may involve multiple immune cell types, such as bovine neutrophils, which respond directly to gastrointestinal nematode O. ostertagi and its derivatives [73] and enhance CD4 T+ cell activation [30]. The presence of neutrophils in secondary lymphoid tissues has been confirmed in healthy humans, mice and cattle [29,30]. Furthermore, neutrophils are found to quickly migrate to draining lymph nodes upon vaccination and challenge from pathogens in mice, which leads to efficient priming of CD8+ T cells [25,26,27,28]. In addition, neutrophils are detected in lymph nodes in parasite infected cattle [30]. Therefore, neutrophils should be able to be present in draining lymph nodes during the first couple of days after infections, which can synergize with APC-derived IL-12 on the activation of CD4+ T cells. 

Neutrophils play a critical role in modulating the adaptive immune response through recruitment of other immune cells, such as DCs, to the site of infection [8]. Increasing evidence suggests that some of them may be directly involved in antigen presentation. MHC II expression by mouse neutrophils may be stimulated by co-culture with CD4+ T cells, which can present antigen to naive CD4+ T cells [8]. Neutrophils from the colons of colitic mice were found to be MHC II+, which can also activate CD4+ T cells [74]. In humans, neutrophils contain a subgroup expressing MHC II that can present antigen to autologous CD4+ T cells [24]. In addition, antigen-presenting neutrophils can be induced in the early stages of lung cancer, an induction that seems to be associated with GM-CSF and IFNγ [75]. Recently, we have found that bovine neutrophils can enhance CD4 T+ cell activation, and a fraction of neutrophils express MHC II [30]. These data all indicate that antigen-presenting neutrophils naturally exist as a subpopulation in humans and animals, which may influence CD4+ T cell activation directly possibly by providing costimulation and secretion of cytokine such as IL-10, or indirectly through interaction with other antigen presenting cells. In the near future, we are interested to investigate how the activation status of neutrophils affects their capability on the activation of bovine CD4+ T cells. In this report, we found that neutrophils can help IL-12 to bypass its requirement for costimulation, which synergistically enhance the activation of CD4+ T cells, and this synergy between neutrophils and IL-12 can be sensitive to inhibitory regulation such as the one triggered by anti-bovine CD28 clone#TE1A (Figure 7). 

## 4. Materials and Methods

### 4.1. Cattle

The Wye Angus herd is a closed herd since 1958 maintained by the Wye Research and Education Center, University of Maryland Experimental Station (Queenstown, MD, USA) [76]. The steers were maintained on the pasture of orchard grass, alfalfa, or clover, and fed with alfalfa, and bailage in winter [77]. At 20 months of age [78], all the steers were processed at a commercial facility (George G Ruppersberger & Sons, Baltimore, MD, USA). Animal Care and Use Protocols were approved by UMD (R-FEB-18-06 and R-JAN-21-02) Institutional Animal Care and Use Committee. All methods were performed in accordance with the relevant guidelines and regulation.

### 4.2. Bovine Neutrophil Isolation

Neutrophils were isolated as previously described [30,73,79] with minor modifications. Briefly, blood was collected from the jugular vein using vacutainers containing EDTA (Becton Dickinson Vacutainer Systems, Franklin Lakes, NJ, USA), and was transferred to 15 mL conical tubes (Fisher Scientific, Pittsburgh, PA, USA) and centrifuged for 20 min at 1000× *g* at 4 °C. Following centrifugation, the plasma, buffy coat, and one-third of the red blood cell pellet were discarded. The remaining cells were suspended in 5 mL ammonium-chloride-potassium (ACK) lysis buffer to remove red blood cells (RBCs). The cell suspension was gently mixed and incubated for 5 min at room temperature (RT). The solution was then centrifuged 10 min at 200× *g* at 4 °C, and the supernatant was decanted. The pellet was washed with 15 mL of calcium- and magnesium-free PBS (CMF-PBS) and centrifuged for 5 min at 850× *g* at 4 °C. ACK treatment was repeated for complete RBC lysis. Cells were then washed twice with 15 mL of CMF-PBS and centrifuged for 5 min at 850× *g* at 4 °C. After the final wash, the pellet was suspended in 1 mL of RPMI-1640 lacking phenol red (Gibco, Fisher Scientific, Waltham, MA, USA), and neutrophil concentrations were measured using the trypan blue exclusion method on a hemocytometer. The purity of isolated neutrophils was around 90% based on FACS analysis, as demonstrated in Supplementary Figure S2.

### 4.3. CD4+ T Cell Isolation 

Inguinal lymph nodes were collected from the cattle in the slaughterhouse. The lymph nodes were cut into 2–3 mm^3^ pieces and digested in 5 mL RP10 medium containing 400 U/mL V Collagenase, 0.1 mg/mL DNase, and 2.5 U/mL hyaluronidase at 37 °C for 2 h [78,80]. The resulting single cell suspension was incubated with FITC-conjugated anti-bovine CD4 (Clone #CC8, BioRad, Hercules, CA, USA), and PE-conjugated anti-bovine CD8 (Clone #CC63, BioRad, Hercules, CA, USA) for 30 min at 4 °C, followed by washing twice with medium. The final suspension contained 2.0 × 10^7^ cells per mL. Sorting was gated on a CD4+/CD8- population in a FACSAria II sorter (BD, San Jose, CA, USA), and the purity of sorted CD4+ T cells was confirmed to be >92%; these were further stained with anti-bovine CD25 (Clone #LCTB2A, Washington State University (WSU), Seattle, WA, USA) to confirm CD25 negative status using a FACSCanto I (BD, San Jose, CA, USA).

### 4.4. Stimulation of CD4+ T Cells

The culture was similar as described previously [30]. Briefly, anti-bovine CD3 (Clone #MM1A, WSU, Pullman, WA, USA) was added to 48-well plates at 10 µg/mL in 250 µL 1X PBS (Hyclone, Logan, UT, USA), as in previous reports [30,81,82]. Anti-CD28 antibody (Clone#CC219, BioRad) was supplemented in culture medium at 5 ug/ml as reported [50]. Recombinant human IL-12 (R & D Systems, Twin City, MN, USA) was supplemented at a final concentration of 20 ng/mL, as reported by [31,32]. Sorted, bovine CD4+ T cells were seeded at 1 × 10^5^ per well in 48-well plates, and autologous neutrophils (from the same cattle) were added to the wells of T cells at 10:1 (1 × 10^6^ neutrophils) ratios as determined in our recent report [30]. Neutralizing Ab against bovine IL-10 (Clone#CC320, BioRad, Hercules, CA, USA) was added to the medium at 10 µg/mL [30,82,83]. Plates were incubated at 37 °C in an atmosphere of 5% CO_2_ for 3.5 days and then analyzed for CD25 and CD62L expression in CD4+ T cells. For IFNγ intracellular staining, a fraction of cultured cells was washed and resuspended in Allos medium supplied with cell activation cocktail (R&Dsystems, Minneapolis, MN, USA), incubated for an additional 4 hours before intracellular staining. All stained samples were analyzed using flow cytometry. 

### 4.5. Bovine Cytokine Array

Supernatants were collected after 3.5-day stimulation under designated conditions. Briefly, the supernatants were centrifugated at 250× *g* for 10 min to further remove debris, and stored immediately at –80 °C before being shipped to RayBiotech (RayBiotech Inc., Peachtree Corners, GA, USA), where the supernatants were tested with Bovine Cytokine Array Q30 (#QAB-CAA-30). 

### 4.6. Flow Cytometry 

Antibodies specific to bovine neutrophils (Clone#CH138A), CD25 (Clone#LCTB2A and Clone#CACT108A), and CD69 (Clone#KTSN7A), CD28 (Clone#TE1A) were obtained from the WSU Monoclonal Antibody Center (Pullman, WA). CD62L-FITC conjugate (Clone#CC32), CD4-FITC (Clone#CC8), CD8-PE (Clone#CC63), CD25-PE (Clone#IL-A111), CD28 (Clone#CC219), IL-10 Abs (Clone#CC320), anti-bovine IFNγ-PE (Clone#MCA1783) were procured from BioRad (Hercules, CA, USA), and all anti-mouse isotype secondary Abs were purchased from Biolegend (San Diego, CA, USA). Manufacturer-recommended concentrations for each Ab were used, typically 1.25–10 µg/mL in 100 µL reaction media.

### 4.7. Statistical Analysis

Statistical analyses were performed with Prism 8 (GraphPad Software, Inc., La Jolla, CA, USA); specific details thereof are provided in the figure legends. Overall, all data have passed the Anderson–Darling normality test. All data were analyzed by one-way ANOVA with Tukey’s Multiple Comparisons Test. Asterisks indicate statistical significance. * *p* < 0.05; ** *p* < 0.01; *** *p* < 0.001.

## Data Availability

Data are contained within the article and Supplemental File.

## Supplementary Materials

The following are available online at https://www.mdpi.com/article/10.3390/pathogens10060694/s1, Figure S1: Gating strategy for CD4+ T cell sorting, Figure S2: Purity examination of purified neutrophils, Figure S3: Gating strategy for CD4+ T cells in coculture with neutrophils, Figure S4: Representative dot plots of IL-4 producing cells.
