# Supplementary material for: Synergistic Activation of Bovine CD4+ T Cells by Neutrophils and IL-12
Source: Pathogens. 2021 Jun 3;10(6):694. doi: 10.3390/pathogens10060694 (PMC8228106; doi:10.3390/pathogens10060694)
Supplement: Supplementary file 1 [file pathogens-10-00694-s001.zip › pathogens-1234863-supplementary.pdf]

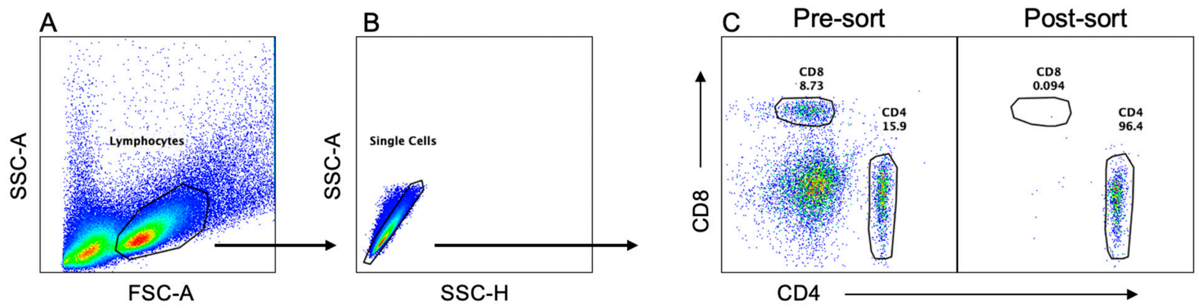

Supplementary Figure S1. Gating strategy for CD4+ T cell sorting. (A) Single cell suspensions of inguinal lymph nodes were stained with anti-bovine CD4 and CD8 antibodies (direct conjugates). CD4 T cell sorting was based on CD4+/CD8- (C) from singlets in lymphocyte population (B). (C) Representative post-sorting purity check.

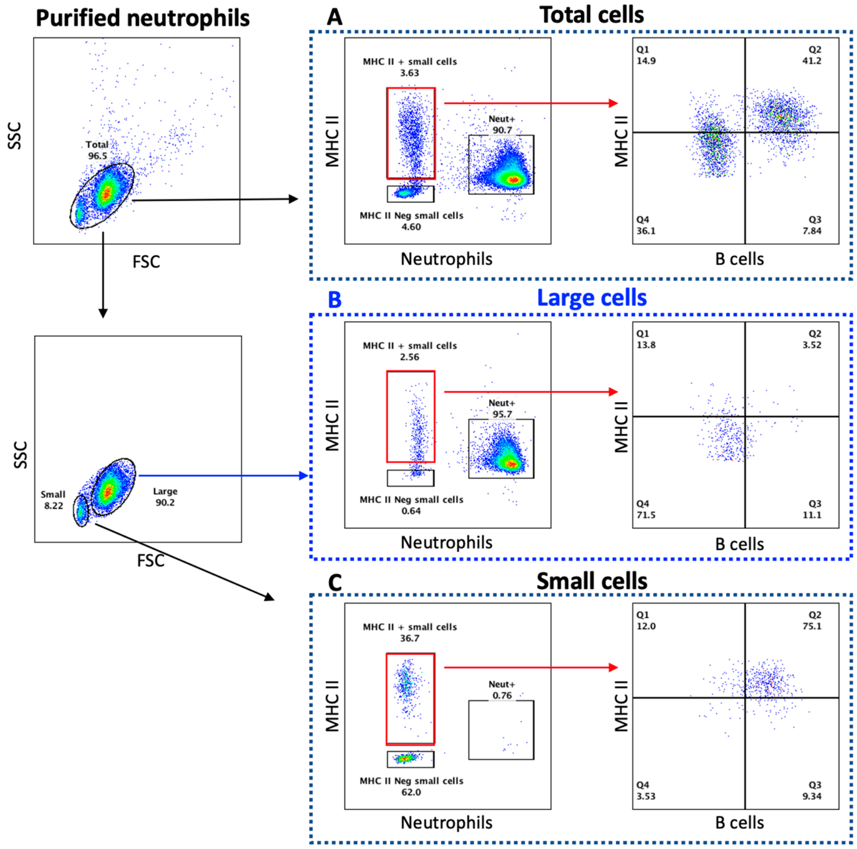

Supplementary Figure S2. Purity examination of purified neutrophils. Bovine neutrophils were isolated from the peripheral blood as described in the Materials and Methods. Purified neutrophils were stained with antibodies for bovine neutrophils, B cells and MHC II, and analyzed on Flow Cytometry. Representative dot plots of purified neutrophils. There were two sub populations, the large and the small, based on SSC/FSC. (A) Total purified neutrophils. (B)-(C) The large (B) and the small (C) sub populations, respectively.

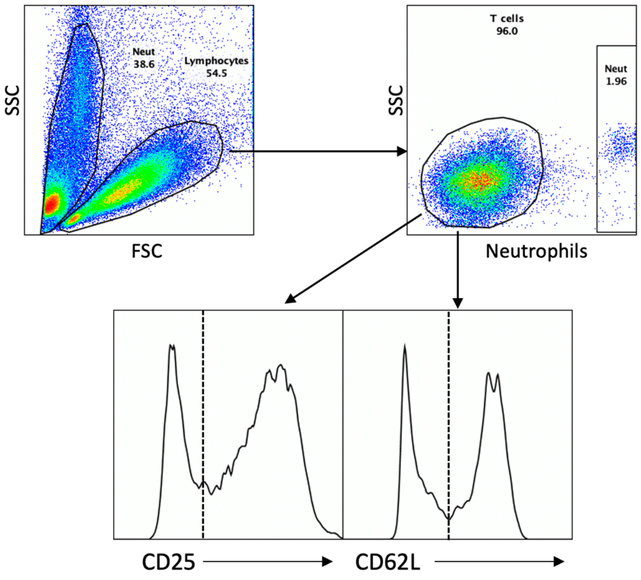

Supplementary Figure S3. Gating strategy for CD4+ T cells in coculture with neutrophils. Sorted CD4+ T cells were stimulated for 3.5 days, and were harvested for staining as described in the Materials and Methods. The analysis of CD4+ T cells was based on neutrophil negative population in lymphocytes.

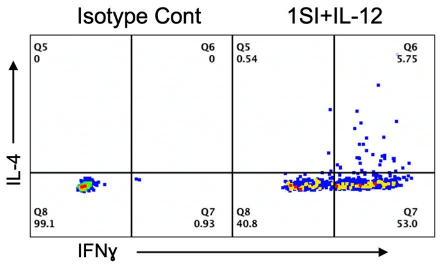

Supplementary Figure S4. Representative dot plots of IL-4 producing cells. Sorted naïve CD4+ T cells were stimulated for 3.5 days, and were harvested for intracellular staining as described in the Materials and Methods. 1SI: αCD3. IL-12: recombinant human IL-12.
